# Hyperparathyroidism: historical milestones and modern therapeutic strategies

**DOI:** 10.1007/s00508-026-02737-5

**Published:** 2026-03-24

**Authors:** Bruno Niederle, Martin B. Niederle

**Affiliations:** 1 Division of Visceral Surgery, Department of General Surgery, Medical University Vienna, Währinger Gürtel 18–20, 1090 Vienna, Austria; 2 Department of General Anaesthesia, General Intensive Care and Pain Management, Medical University Vienna, Währinger Gürtel 18–20, 1090 Vienna, Austria

**Keywords:** Sporadic hyperparathyroidism, Bilateral cervical exploration, Targeted exploration, Chronology

## Abstract

The Viennese Medical School was at the forefront of the clinical understanding of the relationship between parathyroid and bone metabolism. A century ago, F. Mandl (1925) and E. Gold (1927) described two patients clinically (osteitis fibrosa cystica) and biochemically (hypercalcemia, hypercalciuria) with a disease which Gold had already termed (primary) hyperparathyroidism (PHPT) in 1928. The two patients were successfully treated by removing one enlarged parathyroid gland each. After the clinical and biochemical diagnosis was confirmed, surgery was recommended. In 1933, Mandl summarized his rules postulating bilateral cervical exploration, i.e. the macroscopic assessment of all four glands. Only enlarged glands should be removed. Until the beginning of the 1990s, in the hands of highly experienced endocrine surgeons, bilateral neck exploration was the undisputed method of choice (gold standard) with a high postoperative success rate (normocalcemia: 98–99%). Sporadic PHPT is predominantly caused by one hyperactive parathyroid gland. The development of reliable imaging techniques in combination with intraoperative parathyroid hormone (PTH) monitoring to verify the completeness of parathyroid tissue resection led to a change in surgical strategy from extended to limited explorations. The targeted (focused) exposure of the prelocated enlarged gland with the aim to reduce surgical trauma, yet resulting in the same high cure rates, was introduced. For targeted exploration, various endoscopic techniques, cervical or remote access (i.e. skin incision outside the neck), have become available. The more direct the access, the less invasive is the dissection. Surgical techniques minimizing trauma and followed by less pain should be favored. Therefore, the direct open (mini-incision) technique with short neck incision seems to be the new gold standard.

## Introduction

Improvements in surgical training and techniques have made surgical interventions in nearly all organs in various regions possible. Such developments have led to the feasibility of oncologic tumor removal in the neck, at times involving enlarged parathyroids and initially without regard to and knowledge of their function more than 100 years ago.

In 1904, Fritz de Quervain (Basel) [1] removed a cervical tumor which upon histological examination proved to be a parathyroid carcinoma. This case provided the earliest pathologic evidence that parathyroid glands could give rise to a primary malignant tumor. Retrospectively, the tumor was nonfunctioning. Intentional removal of parathyroid neoplasms without documenting their function were also performed by Sir John Bland-Sutton in England some time before 1917 [2] and by Charles H. Richardson in North America in 1924 [3]. The record of early parathyroid tumor excision would be incomplete without reporting the extirpation of a large cervical lesion behind the left thyroid lobe diagnosed clinically as thyroid adenoma in 1926, which surprisingly was interpreted as a parathyroid adenoma on microscopic examination. During follow-up the entity turned out to be a (nonfunctioning) parathyroid carcinoma: Multiple parathyroid implantations were identified in the neck, while subsequent parathyroid tissue recurrences required excision and pulmonary metastases were documented radiologically [4].

A connection between parathyroid function and bone metabolism (and consequently with enlarged parathyroid glands) was earlier suspected by Jakob Erdheim, a Viennese pathologist with great interest and knowledge in parathyroid and bone physiology and pathology [5]. Between 1906 and 1915, Erdheim investigated the parathyroid glands to understand their role in calcium metabolism and bone disease. Using animal experiments and human autopsy material, he examined the ways in which structural changes of the glands correlate with disorders such as tetany, osteomalacia, rickets and osteitis fibrosa. As a result of his studies, Erdheim consistently found an enlargement of the parathyroid glands in osteomalacia and rickets. The degree of parathyroid change correlated with the severity of bone demineralization. In some cases of osteitis fibrosa, he observed marked parathyroid abnormalities, including nodular hyperplasia and tumor-like growth. Therefore, Erdheim was strongly convinced that the parathyroids play a central role in calcium and bone metabolism. He proposed that the documented morphological changes in the parathyroid glands in association with various bone diseases (without distinguishing between osteomalacia, rickets or osteitis fibrosa) may be a compensatory reaction to calcium deficiency or defective bone mineralization.

Earlier, Max Askanazy (Tübingen) [6] and later Friedrich Schlagenhaufer (Vienna) [7] related osteitis fibrosa cystica (OFC) to parathyroid tumors. Schlagenhaufer’s reports were more extensively discussed by Rudolf Maresch (Vienna) [8, 9] and tumors in connection with OFC were meticulously described 10 years after by Siegfried Hoffheinz (Berlin) [10].

## The beginning at the University of Vienna Medical School

The basis of parathyroid surgery applied to control potentially parathyroid hormone (PTH) excess was started in Vienna as an “experiment with a fortunate (and finally at least temporary) successful outcome” in June 1925 [11].

A male patient (Albert J, age 38 years) was admitted to the Department of Surgery II, University of Vienna, and was diagnosed radiologically with severe OFC, first described in detail by von Recklinghausen in 1891 [12] and reported earlier by Friedrich Engel in 1864 [13]. The initially treating surgeon Felix Mandl was unaware of how to help the patient. He was knowledgeable, however, of the reports by Erdheim [5] who interpreted enlarged parathyroids as “compensatory hypertrophy” in (some) patients with osteomalacia and rachitis and who later acknowledged the parathyroid pathology in some variants of bone disease (later understood as OFC) as possibly causal [14]. Mandl first treated Albert J with parathyroid (Collip’s) extract, with no success [15]. Afterwards, again unsuccessfully, he transplanted four fresh human parathyroid glands taken from an accident victim into Albert’s preperitoneal space to replenish the presumed parathyroid deficiency. The patient’s clinical complaints (bone pain, loss of weight, general weakness, increasing immobility) remained unchanged and the bone manifestations (bone cysts, brown tumors) documented by X‑ray imaging worsened. Urine calcium excretion was extremely high, forming white urine precipitates [16].

After these failures, Mandl was now convinced that the “dysfunction” of parathyroids is the “primary” and not the “compensatory” (secondary) cause of the osseous changes revealed radiologically and the severe clinical symptoms. Mandl decided to look for and, upon successful localization, surgically remove a parathyroid tumor although nothing was clinically palpable. With the patient under local anesthesia, bilateral neck exploration (BNE) of the regions behind both thyroid lobes was performed and a left inferior parathyroid tumor alongside three normal-looking parathyroids were localized in July 1925. The firm (25×15×12 mm) tumor, sharply demarcated from its surroundings, was encased in a connective tissue capsule and was sharply dissected successfully from the left recurrent laryngeal nerve [17].

The tissue sections, which were examined by the pathologists Erdheim, Maresch and Anton Priesel, revealed tumoral but no normal parathyroid tissue. The parathyroid cells showed inconsistent sizes and, in some areas, nuclear division patterns and polymorphic cells, morphological indicators suggesting malignancy [18]. As the original tissue blocks were lost in the pathohistological collection of the Clinical Institute of Pathology, Medical University of Vienna, a re-examination applying parafibromin immunohistochemistry [19] or other biomarkers [20], or a reclassification of the original tissue was impossible. Pathologists must be aware of the potential pitfalls that can lead to over-diagnosis of parathyroid carcinoma [21]. The difficulty in distinguishing benign from malignant parathyroid tumors is a problem that remains unsolved as long as no locally invasive tumor is observed during the initial surgery or no metastases develop over time [21, 22]; however, the precise histological report giving many details of the tissue removed during initial surgery was used for reinterpretation (Nicolas Kozakowski, Endocrine Pathology, Clinical Institute of Pathology, Medical University of Vienna; personal communication). According to current histopathological classification [22], the formerly removed parathyroid tumor may have corresponded in many aspects (including the autopsy findings; [no local recurrence, no metastasis]) with what is now called atypical parathyroid adenoma.

Postoperatively Albert’s physical condition improved dramatically. Chronic bone pain disappeared. Bone X‑ray revealed significant improvement. Urine calcium excretion was documented preoperatively and postoperatively. Having reached a high level prior to surgery, the urine cleared within days and calcium excretion dropped to normal. The preoperatively known presence of nephrocalcinosis remained unchanged while kidney stones became symptomatic [18, 23]. Albert J’s successful postoperative clinical course confirmed the former proposal made by Schlagenhaufer [7] to look for and remove enlarged parathyroids in patients with severe radiologically confirmed generalized OFC even without clinically palpable cervical lesions. Edheim’s theory was refuted.

The second patient with severe OFC was admitted to the Department of Surgery I, University of Vienna, two years after surgery performed on Albert J, and was seen by Ernst Gold [24]. Gold was influenced by the highly promising and steady improvements of Albert J’s postoperative clinical course, which Mandl continuously documented [18]. Gold performed a BNE in Barbara F (age 54 years) in July 1927. He localized and removed an enlarged right superior parathyroid gland which was classified histologically as parathyroid chief cell adenoma [25].

Together with Hermann Barenscheen, a specialist in applied medical chemistry, the preoperative and postoperative calcium levels and urine calcium excretion were for the first time systematically revealed in detail. “Cure”, i.e. normocalcemia and normal urine calcium excretion, was documented four weeks postsurgery [25]. Critically analyzing the preoperative and postoperative laboratory findings, this was the first compilation of laboratory data of this kind in the literature. Gold concluded that “like exogenously administered PTH, the enlarged and therefore hyperfunctioning parathyroid tumour may cause, as a biochemical manifestation of its over-activity, increased serum calcium (hypercalcaemia) and increased urinary calcium excretion (hypercalciuria) through mobilisation of calcium from bone”. For the first time in the literature, he proposed to summarize these clinical and biochemical findings as “(primary) hyperparathyroidism” (PHPT) [25].

At that time, James B. Collip [26] prepared parathyroid extracts and recommended the therapeutic use for hypocalcemia but the hormone itself had not yet been purified.

In the first patient, disturbed parathyroid metabolism was suspected on the basis of severe osseous manifestations on X‑ray and the patientʼs enormous urine calcium excretion. In the second patient, hypercalcemia and hypercalciuria were also documented biochemically beside severe OFC radiologically. The functional connection between the parathyroid glands and the mobilization of calcium from bone was now meticulously confirmed biochemically and for the first time in the literature [25].

Albert J developed the first biochemical and clinical signs of recurrence six years after successful initial surgery as documented in detail [27]. During bilateral re-exploration, no parathyroid tumor was found in typical or atypical cervical locations, not even after bilateral subtotal thyroid resection. A normal parathyroid was harvested from each side. Mandl suspected a parathyroid neoplasm in a location inaccessible to surgical exploration [28]. As expected, no changes were identified in the laboratory findings and in the clinical symptoms. Urine calcium excretion and the blood calcium levels remained elevated, phosphate persistently decreased. In an unexpected way, no symptoms of hypocalcemia were documented postoperatively, although a total of three parathyroids had been removed during the two neck explorations [27, 28]. By definition, PHPT persisted and the patient died from uraemia 3 years later (11 years after the first surgical intervention). Autopsy revealed extensive cystic fibrosis of the pelvic bones and both femurs. Brown tumors were described in both femurs and in the clavicle with decalcification and fractures in the proximal and distal parts of the spinal column. Bilateral kidney stones and bilateral hydronephrosis and nephrocalcinosis were documented. Very disappointingly, neither a local recurrence nor parathyroid metastases (locoregional lymph nodes, lung, liver as a possible sign of a malignant parathyroid disease) or pathologically altered parathyroid tissue in an ectopic position were found. For this review the original autopsy report was retrieved from the archives of the Department of Pathology, Medical University Vienna. It notes that no tumorous parathyroid tissue was found “… in the paratracheal space, the mediastinal or retroperitoneal regions.” However, the “aortopulmonary window,” a very rare ectopic parathyroid location in the middle mediastinum, not known as a possible ectopic location at that time [29] was not specifically investigated and is not specifically mentioned in the autopsy report. Maybe this was the location of the “undetected” parathyroid adenoma. Assuming a thorough autopsy (performed in Mandl’s presence), the cause of the recurrence unfortunately remains unknown [30].

As early as 1933, Mandl [31] summarized the Viennese experiences and those with 55 surgically treated patients published in the literature. He analyzed in detail and established for the first time step-by-step instructions for systematic parathyroid exploration as the standard of care (Mandl’s rules; Table 1) before the advent of modern localization and hormone assays which, slightly modified [32, 33], influenced later generations of endocrine surgeons. These instructions remained valid at least until the end of the 1990s [34] and are still applied today in special situations (unlocalized disease; primary parathyroid hyperplasia; involvement of PHPT in hereditary endocrine tumor syndromes; [35]). The specific manifestation of PHPT and its diagnosis and treatment in connection with the currently known hereditary endocrine tumor syndromes are not discussed here.

**Table 1:** Mandl’s rules: step-by-step instructions to explore patients with biochemically confirmed sporadic primary hyperparathyroidism [31]

Bilateral cervical exploration to identify all four parathyroid glands is mandatory
Only enlarged glands should be removed
If localization is unsuccessful in typical locations: careful search for ectopic glands (e.g., in the mediastinum within thymic tissue, along the esophagus) is helpful
Preservation of normal parathyroid tissue to avoid permanent hypoparathyroidism is recommended
Attention to the recurrent laryngeal nerve and vascular structures (especially to normal parathyroids) to minimize permanent hypocalcemic and other complications is necessary

**Table 2:** Clues to the diagnosis of hyperparathyroidism in the first 343 cases at the Massachusetts General Hospital [75].

Clue	*n*	%
Bone disease	80	23.3
Renal stones	195	56.9
Peptic ulcer	27	7.9
Pancreatitis	9	2.6
Fatigue	10	2.9
Hypertension	6	1.7
Mental disturbance	3	0.9
Central nervous system signs	7	2.0
Multiple endocrine abnormalities	3	0.9
Lump in the neck	1	0.3
Asymptomatic	2	0.6

These comprehensive recommendations were intended to improve diagnosis, maximize surgical success and minimize postoperative complications.

Hypercalcemia in combination with hypercalciuria facilitate a clear distinction of generalized OFC (hyperparathyroidism; HPT) from other pathological bone changes, such as localized OFC, osteomalacia or Paget’s disease, which were not yet clearly differentiated at that time [36].

Additionally, hypophosphatemia and hyperphosphaturia were later identified in the majority of patients and added as biochemical characteristics of PHPT [37, 38].

Surgery will be successful only when PHPT is definitively confirmed biochemically, being the only way to definitively achieve sustained normocalcemia (i.e. cure), thus treating PHPT, at that time a life-threatening disease [39].

In the course of systematic extended BNE, all four parathyroid glands have to be identified. Normal and pathologically altered lesions must be clearly distinguished macroscopically. Consistently, only enlarged glands (predominantly one enlarged gland, in isolated cases two enlarged glands) should be removed by “selective parathyroidectomy (SP)”. Preservation of normal parathyroid tissue and of its blood supply is mandatory to avoid permanent hypoparathyroidism, and a meticulous preparation of all cervical strictures is necessary, especially to avoid any damage to the recurrent laryngeal nerves [31].

In cases of doubt, parathyroid organ diagnosis must be confirmed by frozen sections. Enlarged glands are usually separable from the thyroid gland or any other neighbouring tissue. To achieve “cure”, the surgeon has to ensure that the parathyroid glands (normal and abnormal) are localized during surgery, taking account of anatomical variations and systematically exploring typical and atypical parathyroid locations. The identification of the parathyroid glands during meticulous, atraumatic exploration can be challenging due to their variable numbers [40] and anatomical locations [41–43].

In individual cases, especially if a parathyroid cannot be localised in the typical location, a careful search for ectopic glands (e.g., behind the clavicle and sternum in the mediastinum within thymic tissue, in the thyroid or along the esophagus) must be performed [31, 33, 44].

The knowledge of embryonic migration of the parathyroids, as well as awareness of their typical and atypical (ectopic) positions and vascular supply are therefore essential for successful surgery. The anatomical orientation along the recurrent laryngeal nerve and the inferior thyroid artery as key landmarks improves the intraoperative detection rates of parathyroid glands and parathyroid tumors. Anatomical variability is higher for the inferior than for the superior parathyroid glands. In the collected series from the literature, 25% of adenomas were not found in a typical position [31].

Unpredictably, tetany may occur immediately (on the first day) or sometimes, with a slight delay of up to 10 days after surgery in patients with extensive osteitis fibrosis and impending transient (hungry bone syndrome; HBS) or permanent hypopcalcemia as a consequence of calcium imbalance caused by overextensive resection of parathyroid tissue. Undetected calcium levels below 8 mg/dl (2.0 mmol/l) have led to life-threatening complications. In the literature review, Mandl [31] documented a 7.3% mortality rate, with most deaths attributable to tetany. He thus highly recommended generous and adequate calcium substitution based on short-term blood calcium monitoring to prevent severe life-threatening uncontrolled hypocalcemia.

As a consequence of his own experience and those reported in the literature, Mandl recommended “calcium screening” in patients with radiologically suspected OFC and, once diagnosis was confirmed, early surgery to correct parathyroid dysfunction before severe bone changes occur. He also advocated prophylactically applying calcium and PTH extracts (intravenously in severe OFC) for 3 weeks to prevent severe tetany post-surgery. Thereafter he proposed consistent monitoring of calcium levels after reducing the supplementation not to overlook prolonged hypocalcemia within HBS [31].

By definition HBS [45], a serious adverse effect of parathyroidectomy, refers to rapid, profound and prolonged hypocalcemia associated with hypophosphatemia and hypomagnesemia and is exacerbated by suppressed PTH levels, which follows parathyroidectomy in patients with severe PHPT and preoperative high bone turnover. Severe hypocalcemia is believed to be due to increased influx of calcium into bone caused by the sudden removal of the effect of high circulating PTH levels on osteoclastic resorption. This process results in a reduced activation frequency of new remodelling sites and a decrease in remodelling space, although there is no good documentation for this. The syndrome is reported in approx. 25–90% of patients with radiological evidence of hyperparathyroid bone disease vs. only 0–6% of patients without skeletal involvement. Treatment is aimed at replenishing the severe calcium deficit to resolve hypocalcemia, which may persist for a number of months after successful surgery.

As shown recently [46], normal parathyroid metabolism was only documented in 32.7% of the patients on the first day post-surgery. This is of great clinical importance to all physicians responsible for the postoperative care of patients with PHPT. Patients must be followed biochemically for at least 6 months to document surgical success. The necessity of calcium and vitamin D3 substitution cannot be safely predicted preoperatively or postoperatively before days 5–7 following surgery without serial calcium and PTH measurements. As an efficient and cost-effective approach, and in order to carefully monitor for unpredictable HBS and (temporary) hypoparathyroidism, a routine 8 weeks of calcium and vitamin D3 substitution according to PTH levels on postoperative day 1 has recently been recommended [46].

## Further experience in other institutions—Clinical manifestations and symptoms

Hyperfunction of the parathyroid glands and its clinical impact on the human body were described almost simultaneously by surgeons in Europe (Vienna General Hospital, Vienna Medical School; 1925) and by internists in North America (Bellevue Hospital in New York City, Massachusetts General Hospital in Boston; 1926). At the beginning, the two groups knew nothing about each other and were not aware of the clinical and laboratory findings of their transatlantic peers.

The first male patient (Charles M, age 30 years) with severe OFC and suspected hyperactivity of the parathyroid bodies was documented in New York City (USA) by Eugene F. Dubois, revealing hypercalcemia and hypophosphatemia during the initial metabolic investigations in 1926 [38]. The biochemical diagnosis was validated and the diagnosis confirmed in Boston by more detailed metabolic studies of calcium and phosphate metabolism [47].

Based on the laboratory findings, surgery was discussed. It was decided to look for a parathyroid tumor and the cervical exploration was performed at the Surgical Department of Massachusetts General in 1926 [48]. During a 1-month interval, a left and right neck exploration with removal of one normal parathyroid gland on each side was performed. The disturbance of parathyroid metabolism persisted after the two unsuccessful cervical explorations and five more operations followed. During the seventh transsternal procedure, encouraged by the patient himself (!!), a parathyroid adenoma pendant from a pedicle with its origin in the neck was partly removed by anterior mediastinotomy left of the inferior caval vein [49]. Subsequently, 10% of the tumor was left in situ, turned up on its pedicle and sewed in a superficial position in the region of the sternal notch to minimize or avoid expected severe tetany; however, the postoperative level of serum calcium fell rapidly and signs of tetany emerged on the third day. The patient’s recovery from the operation was satisfactory and his general condition improved but one month after surgery one of the kidney stones became lodged in the patient’s ureter and required surgical intervention. This was followed by a series of complications and six weeks after the removal of the parathyroid tumor the patient died of renal failure [50].

Generalized OFC was suspected and diagnosed radiologically in Charles M and extensive metabolic investigations were started. The preliminary biochemical results were presented before the Interurban Clinical Club, New York, in 1927 (1 year after Mandl’s publications [17]). With a delay the results of the studies confirming parathyroid hyperfunction were published in detail in 1930 [38] and did not discuss Mandl’s [17] and Gold’s [25] experiences.

Without knowledge of the extended metabolic studies conducted in Charles M, three reports, the first by Gold [25] and then subsequently by two Americans [51, 52] were published in parallel on mineral metabolism in generalized OFC and yielded identical laboratory results. The clinical details of a 21-year-old man with biochemically confirmed PHPT and clinically increasing weakness, difficulties with walking and bending his lower extremities and lumbar lordosis were reported by Boyd, Milgram and Stearns [53].

In all four patients described in the literature up to 1930, single parathyroid tumors were removed during one cervical exploration with clinical improvement following surgery. The tumorous tissue removed by Wilder was finally classified as “malignant adenoma” [54].

At that time, “OFC” (termed “hyperparathyroidism” in the literature, as recommended by Gold [25]) was thought of as a clinically distinct generalized skeletal disease, seen to be associated with bone pain, characteristic X‑ray findings such as cystic bone tumors, pathological fractures or gross skeletal deformities associated with kidney and ureteral stones (usually bilateral), which was caused by alterations of calcium and phosphorus metabolism due to a parathyroid adenoma [49]. Some patients were also documented with polydipsia, polyuria, weakness, loss of strength, appetite, weight or constipation [49]. These symptoms are now summarized and well-known under the term “hypercalcemia syndrome”, also extensively documented in the first Austrian patients Albert J and Barbara F.

The initial foundations for the understanding of the physiology, pathophysiology and surgery of the parathyroid glands were published by representatives of the Vienna Medical School (the pathologists Erdheim and Maresch; surgeons Mandl and Gold). Following these foundations stimulating some others, mainly the team in Boston, endocrinologist Fuller Albright^1^, pathologist Benjamin Castleman and surgeons Edward Delos Churchill and Oliver Cope, rapidly became pre-eminent in this field.

The Boston group demonstrated that renal and ureteral stones may be the first and at times dominating clinical manifestations of the disease [55]; however, at the same time, Mandl reinforced the possible causal link between parathyroid overactivity and renal stone formation in an experimental rat model [56]. Patients could have PHPT without suffering from bone disease, and others could have bone disease without renal stones. It was obvious that both bone and stones diseases in combination or alone were clinical manifestations of the disease, not primary findings. In a series, Albright [37] documented that many patients with PHPT were identified only because their serum calcium and phosphate were measured when they presented with kidney stone disease (stone disease) rather than obvious bone disease. The authors supported screening for PHPT in kidney stone patients by showing that measuring calcium in stone formers can reveal an underlying parathyroid pathology and can prevent renal complications, as documented in the first patients Albert J and Charles M: Patients with kidney stones, some with mild bone decalcification but many without any evidence of bone disturbance, emerged to be the largest group.

Some patients complained of abdominal symptoms, which disappeared promptly when PHPT was corrected. Peptic ulcer [57, 58] and various types of pancreatitis [59] were recognized as secondary clinical symptoms to an underlying PHPT (Table 2).

Bone disease, renal stones and kidney calcification were common manifestations, while peptic gastric, duodenal ulceration and pancreatitis were rare symptoms of PHPT. Keeping these observations in mind, it was recommended that patients with any type of pancreatitis should be biochemically screened for possible HPT in the same way as those with characteristic findings of bone disease, renal stones or peptic ulcer disease.

There are only few reports on the metabolic complications [60] and the long-term clinical results of parathyroidectomy for PHPT [61]. The long-term clinical courses of 212 “cured” (normocalcemic) patients were analyzed for 1–25 years (mean 6.8 ± 5.4 years). Preoperatively, 181 patients (85%) were classified as having typical renal and osseous manifestations or gastrointestinal symptoms, 22 patients (11%) as having minimal symptoms (only hypertension, diffuse osteopenia or manifestations of the hypercalcemic syndrome such as depression, loss of concentration, confusion, nausea, vomiting, polyuria, polydipsia and recurrent abdominal discomfort without organic explanation), and 9 patients (4%) as having no symptoms (asymptomatic patients). Although the formation of kidney stones was stopped in 91% of the patients, deteriorated renal function and hypertension were seen in patients with symptoms (14 and 8%, respectively) and in patients with minimal symptoms (6 and 15%, respectively). Renal function changes and hypertension were unpredictable despite normalization of hyperactive parathyroid metabolism, and they had decisive outcomes: of the patients 7% died of uremia or the consequences of hypertension (stroke). Large, multiple bone lesions healed functionally and were of no prognostic significance. In the majority of patients with PHPT symptoms, gastrointestinal symptoms disappeared postoperatively, but two patients who had had no preoperative gastrointestinal complaints died of acute pancreatitis. Almost all symptoms of the hypercalcemia syndrome disappeared immediately and permanently in symptomatic and minimally symtomatic patients. Neither deterioration of renal function nor elevation of blood pressure were observed postoperatively in “cured” patients who had shown no symptoms of PHPT preoperatively. Even in these patients, immediate surgical treatment may have avoided the complications of chronic renal failure or hypertension. As soon as organic manifestations, even in mild forms, have been established, it seems impossible to predict the course and to prevent an unfavorable clinical outcome [61]. It can be concluded that the earlier the metabolic disorder is diagnosed and treated, the better the long-term outcomes prove to be. Prolonged chronic hypercalcemia can cause irreversible damage to organs such as kidneys or bones.

The PHPT was considered a rare metabolic disorder until the mid-1960s. The increase in incidence was driven by routine serum calcium determinations [62] and by the development of immunoassay techniques for measuring PTH and enabling a major differential diagnostic refinement in the case of hypercalcemia [63]. The techniques were further improved to the point where they also became available for widespread clinicsl use in established laboratories [63].

A highly sensitive, two-site immunoradiometric assay (IRMA) for human PTH that is specific for the intact, secreted, biologically active 84-amino-acid peptide was introduced by Nussbaum et al. [64]. The assay readily measures concentrations of PTH in all healthy subjects and distinguishes these values from low or undetectable PTH values observed in clinical situations, in which PTH secretion is expected to be suppressed. The sensitivity, specificity and rapid turnaround time of this two-site IRMA further improved the laboratory evaluation of patients with calcium metabolic disorders [64].

## Incidence of PHPT in Austria and practical advice for diagnosing PHPT

The number of patients detected with PHPT increased dramatically and PHPT has now become the third most common endocrine disorder [65].

In a prospective study in Lower Austria, serum calcium determinations over an 8‑year period in 45,217 hospitalized patients revealed sustained hypercalcemia in 124 (0.3%) subjects. Further clinical and biochemical investigations established PHPT in at least 40 (32%) patients, a PHPT incidence of 89.6 ± 41.5 per 100,000 patients per year. The incidence of PHPT in males and females was 31.5 and 124.2 per 100,000, respectively. The highest incidence was found in females older than 66 years of age (268.8 per 100,000): 19 (48%) patients showed classical (renal, bone) manifestations or gastrointestinal symptoms, 14 (35%) patients were minimally symptomatic (osteopenia, hypertension, symptoms summarized under the term “hypercalcemic syndrome”) and 7 (17%) patients were asymptomatic. The incidence and symptomatology of PHPT in Austria was nearly the same as in other countries [66].

Because of the multiple actions of PTH in the body, PHPT can cause debilitating symptoms, yet it too often remains clinically undiagnosed. Routine calcium determinations are very important: PHPT must always be considered in the differential diagnosis, especially in minimally symptomatic patients. The clinical presentation of PHPT has changed dramatically in recent years, documenting a significant increase in minimally and asymptomatic patients [67].

There are many causes of hypercalcemia [68]. Therefore, the differential diagnosis of hypercalcemia, the definitive diagnosis of PHPT and, by consequence, parathyroid surgery is mandatory. It is very important to exclude tumor-induced hypercalcemia (as the second most frequent cause of hypercalcemia) and familial hypocalciuric hypercalcemia (FHH). Hypercalcemia is the key laboratory parameter of PHPT. In combination with elevated PTH, repeated elevated serum calcium levels in combination with low urinary calcium excretion assist in correct biochemical diagnoses of PHPT.

The biochemical differential diagnosis of hypercalcemia should always include PTH, creatinine, Estimated Glomerular Filtration Rate (eGFR), 25-hydroxyvitamin D and urinary calcium excretion measurements [69, 70]. Calcium excretion in the 24‑h urine sample or the calcium/creatinine clearance ratio help to distinguish between PHPT and FHH. Patients with FHH are not candidates for surgery [71].

## Treatment options of PHPT

### Surveillance

Due to the widespread use of routine calcium measurements, the number of patients with incidentally discovered hypercalcemia is on the increase. In parallel, a shift has occurred from classical “symptomatic” to clinically “mildly” and “asymptomatic” patients [67]. Undoubtedly, the “symptomatic” patient is a candidate for surgery; however, there is an ongoing discussion as to whether patients with “mild” or those “without” symptoms should undergo surgical treatment [69]. Many “asymptomatic” patients screened for the presence of nephrolithiasis and/or vertebral fractures will be reclassified as having symptomatic disease in further investigations, and many members of the “mild” and “asymptomatic” group improve in terms of quality of life. Watchful waiting requires strict patient discipline, including consistent laboratory tests, at least yearly monitoring of calcium and renal function and it must exclude possible organ manifestations in bone and in the kidneys. Surveillance entails corresponding costs for the healthcare system.

Patients who are unfit for surgery or absolutely do not meet the criteria for surgery [69] are candidates for pharmacotherapy including calcimimetics (such as cinacalcet). These drugs are agonists of the calcium-sensing receptor (CaSR) that act as allosteric modulators to increase the sensitivity of CaSR to calcium stimulation; hence they can suppress PTH secretion and normalize serum calcium; however, calcimimetics do not alter bone mineral density and the effects on fracture risk and quality of life are unknown. Cinacalcet for hypercalcemia and anti-resorptive therapies for osteoporosis may be an option [69].

Estrogen replacement (hormone replacement therapy, HRT) can lower calcium concentrations and have beneficial effects on bone mineral density by acting on the skeleton rather than the parathyroid glands. On account of the associated risks of HRT, however, it should not be used solely for treating PHPT. Selective estrogen receptor modulators (SERMs), such as raloxifene, significantly decrease calcium concentrations with presumably the same mechanism of action seen with estrogen replacement, but the evidence is limited.

Bisphosphonates improve bone mineral density and can suppress PTH-mediated bone resorption, but do not improve serum calcium or PTH levels in the long term. Furthermore, when compared to surgery, bisphosphonates do not show an improved fracture rate. They are occasionally used as a “bridge to surgery” for the temporary reduction of exceedingly high calcium levels (emergency situations) in patients awaiting surgery.

### Parathyroid surgery

In the course of BNE,(definition see Table 4), it may be a problem to assess the morphological nature of the parathyroids. When a gland is found, it must be carefully dissected so that its size can be evaluated; however, excessive dissection can destroy the glandʼs blood supply resulting in hypofunction. A parathyroid may be considered “enlarged” on the basis of macroscopic appearance alone and not on its histology. Localizing and visualizing an enlarged parathyroid gland do not imply that it must be a single parathyroid adenoma. At least two glands, ideally all four glands, must be exposed and assessed macroscopically by BNE to determine with a high degree of probability whether a “single gland disease” (SGD; single adenoma, carcinoma) or “multiglandular disease” (MGD; double adenoma, four-gland disease) is present.

However, the macroscopic differentiation between SGD and MGD can be macroscopically (and even by frozen section) challenging. Yet as the surgical strategy must be adapted accordingly, it is of utmost importance.

In 1934, Churchill became aware of patients with a generalized enlargement of all four parathyroid glands which were, unlike adenoma, classified histologically as “(water)clear cell hyperplasia (WCCH)” [72, 73]. Another major contribution from the Boston group developed when it described “primary chief cell hyperplasia (CCH)” of the parathyroid glands as a new entity of PHPT [74]. The Boston group [75] documented 76.7% and 3.8% patients with single or double adenoma, respectively, and 15.2% with primary (chief or water-clear) parathyroid hyperplasia (Table 3).

**Table 3:** Causes of hyperparathyroidism in the Massachusetts General Hospital; series of 343 cases (1930–1965) [75]

Cause	*n*	%
*Neoplasia*		
Single adenoma	263	76.7
Double adenoma	13	3.8
Carcinoma	15	4.4
*Primary hyperplasia*		
Chief cell	37	10.8
Clear cell	15	4.4
Total	343	100.0

While in patients with one (or rarely two) enlarged parathyroid glands, these lesions are removed after BNE by SP, there is the need to excise three or more glands in patients with any variant of diffuse parathyroid hyperplasia, leaving behind a small piece of one gland with an intact blood supply (3½ or subtotal parathyroidectomy; SPTX) [74, 76]. Preoperative parameters do not enable the prediction of whether PHPT is caused by a single or multiple enlarged glands [55, 77, 78].

Therefore, SPTX requires experience in parathyroid surgery. While removing solitary (or two) enlarged gland(s) is technically simple in general, SPTX may prove difficult as it requires considerable experience in the macroscopic assessment of and in the reduction of gland size. A viable remnant of parathyroid tissue equivalent to a normal-sized gland (or estimated at one quarter to one half of an enlarged gland) should be left in order to maintain normal parathyroid metabolism.

The surgical technique determines the postoperative outcome. Postoperative metabolic problems can arise if an over-excessive or an insufficient amount of parathyroid tissue is resected, resulting in permanent hypo or persistent HPT, respectively [79]. Several authors have proposed BNE with SPTX instead of SP in all patients with PHPT [80]. Their data suggest that PHPT may recur in up to 30% of patients treated by SP of only enlarged glands. This recurrence was attributed to undetected primary CCH as the pathology in up to 65% of all patients [81, 82].

Arguing the high rate of permanent hypocalcemia, others have warned against unnecessary too extended parathyroid resections [83]. In their study, Edis et al. [84] addressed the question whether the recent trend toward more radical parathyroid surgery is justified comparing the surgical outcomes in 3 groups of 50 patients undergoing surgery for PHPT. One group of patients was operated on by surgeon A who applied a conservative approach (extended BNE with selective removal of grossly enlarged glands only, with or without biopsy of one normal-sized gland). A second group was treated by surgeon B, who used a more liberal approach (extended BNE with routine removal of at least two glands, removal of three and subtotal resection when more than one gland was enlarged and liberal use of biopsy identification). Symptomatic hypocalcemia requiring treatment occurred in 24% of patients after liberal neck exploration, as compared to 4% in the conservatively treated group. The liberal approach did not yield a higher cure rate. A third group of 50 patients was operated on by surgeon B using the conservative approach. The incidence of postoperative hypocalcemia was reduced to 2%; one patient remained hypercalcemic. Symptomatic hypocalcemia, even if temporary, represents a significant morbidity. A conservative approach to neck exploration in patients with PHPT was recommended because it was associated with a very low incidence of temporary postoperative hypoparathyroidism (2–4%) and a high cure rate (99%).

Following Mandl’s rules, BNE with SP (without biopsy of normal-looking glands) remain the surgical strategy of choice in the majority of large centers, with excellent long-term results documenting normocalcemia between 92–98.6% after initial exploration [34, 85–89].

The definition of terms and surgical techniques used in literature and in this manuscript are clearly listed in Table 4.

**Table 4:** Definition of terms and explanation of techniques used in the manuscript (in literature)

Technique	Abbreviation—Access
**Extended exploration**	
Extended bilateral exploration	BNE—Conventional (open) approach through a transverse cervical incision (Kocher incision) > 30 mm allowing exploration of both sides and identification and assessment of all four glands
**Limited exploration**	
Unilateral exploration*	UNE—Conventional (open) approach through a transverse cervical incision (short Kocher incision) around 30 mm; Identification and assessment of two (one enlarged hyperfunctioning and one normal) glands of one side; with/without biopsy of the normal-looking gland
*Targeted, focused exploration*	TE, FE—Selective exposure of the prelocalized parathyroid gland
Open (mini-incision) technique*	OMIP—Conventional (open) approach through a “short” < 30 mm transverse (paramedian/lateral) cervical incision (very short Kocher incision)
Video-assisted technique*	VAP—Endoscopy-assisted approach through a small 15–20 mm cervical incision used to remove a prelocalized parathyroid gland
Endoscopic technique	EP—Totally endoscopic approach
	- Cervical access* - Remote-access: axillary approach, anterior chest approach, bilateral breast areola and ipsilateral axillary (BBIA) approach, bilateral axillo-breast approach (BABA) and the postauricular and axillary approach (PAA) Transoral exploration via a vestibular approach
**Selective parathyroidectomy**	SP—Recommended during all explorations: exposure and removal of only one (or in selected patients more enlarged glands); preoperatively or intraoperatively suspected as hyperfunctioning; no biopsy of normal-looking glands
**Transcervical thymectomy (unilateral/bilateral)**	(Transcervical blind) mobilization and removal of the thymus remnant (and SP) through a deep cervical short Kocher incision
**Transsternal mediastinal exploration**	Open exploration with removal of the thymus remnant (and SP) through a partial or total median sternal split
**Transthoracic exploration**	Open exploration—Preferably transthoracic endoscopic removal of the thymus along with SP

Surgery with “direct” access: targeted (TE), focused exploration (FE) resulting in selective exposure of the pre-localised parathyroid gland and selective extirpation of this gland, i.e. selective parathyroidectomy (SP), with the skin incision in the neck are considered minimally invasive procedures (*), i.e. minimally invasive parathyroidectomy (MIP)—they can be performed openly (by a mini-incision, video [endoscopic]-assisted or total endoscopic). OMIP may be performed in local anesthesia, MIVAP in cervical block anesthesia

Surgery with a “remote-access” (uni-bilateral axillary approach, anterior chest approach). Bilateral breast areola and ipsilateral axillary (BBIA) approach, bilateral axillo-breast approach (BABA) and postauricular and axillary approach (PAA); transoral exploration via a vestibular approach allows targeted, focused exploration with SP with a skin incision (scar) outside the neck, generally using two or more trocars through short incisions. There are long “working canals” from the incision area(s) (axilla, region above the areola, mouth) to the neck resulting in extensive soft tissue trauma—these techniques are no longer called minimally invasive

**Table 5:** Intraoperative parathyroid (IOPTH) monitoring: yes or no?

Exploration/technique	IOPTH yes/no
*BNE open*	No
*BNE VA/EN*	No
*UNE open/VA/endoscopic with MIBI/US*	Yes/no
*Targeted, focused*	
With MIBI and US concordant	No/yes
With MIBI and/or US discordant	Yes
With MIBI and/or US negative	Yes

*BNE* (extended) bilateral neck exploration; *UNE* (limited) unilateral neck exploration; *VA* video-assisted; *EN* endoscopic; *MIBI* 99mTc-sestamibi scintigraphy; *US* ultrasonography; *IOPTH* intraoperative parathyroid hormone quick assay

The underlying morphological cause of the metabolic disorder in these centers was, as expected, a solitary parathyroid adenoma. A minority of surgeons thus believed that a limited unilateral exploration (UE) should be sufficient if an abnormal and a normal gland were identified on the side first explored documenting SGD making it unnecessary to explore the contraleral side [90–92].

To confirm the macroscopic diagnosis of SGD and to secure the decision to perform a UE procedure, the density test and special intraoperative oil red O staining were recommended, as both are able to increase the difference between hyperfunctioning and uninvolved parathyroid tissue [93, 94]. The principle of limited UE and SP (with biopsy of the unilateral normal-looking gland) in conjunction with the intraoperative oil red O staining technique offered a more reliable perioperative distinction between SG and MGD. It assisted in reducing operation time, decreasing the complication risk, reducing early hypocalcemia and providing more favorable technical conditions for re-operation, if necessary [95]. In 43/102 (42.5%) of the patients in whom the abnormal gland was found on the side explored first, unilateral parathyroidectomy was performed on that side, avoiding exploration of the contralateral side. In 45/102 (44.1%) patients in whom normal glands were found on the side explored first, unilateral parathyroidectomy was performed on the contralateral side. As the abovementioned principle could not be achieved in 14/102 (13.7%) patients, other types of operations were performed. Of the patients who had atypical surgery two had a permanent need for vitamin D in order to maintain an adequate serum calcium level. Compared to more extended explorations, the long-term results were equivalent as based upon the idea of performing UE with selective parathyroidectomy whenever intraoperative oil red O staining on frozen sections excluded multi-glandular involvement as a cause for PHPT [91].

The introduction and consistent application of radiological and nuclear medicine techniques, such as ultrasound (US) [96, 97] and thallium-201/technetium-99 m (Tc/Tl) subtraction scanning [98] facilitated the morphological and functional imaging of enlarged parathyroid glands in preoperatively planning the surgical strategy (limited or extended exploration) in patients with biochemically confirmed PHPT.

The Tc/Tl isotope subtraction scanning was used routinely as a preoperative localization investigation in 90 patients with PHPT [99]. The scintigram demonstrated a single focus in 48/90 (53.3%). Scan-directed UE with SP and biopsy of the ipsilateral normal gland were applied while the remaining 42 individuals had standard BNE. The difference in operating time for patients who underwent UNE and BNE was statistically highly significant (71 min vs. 97 min, *p* ≤ 0.001). At a mean follow-up of 16.8 months, no patient who had a UNE performed for solitary parathyroid adenoma demonstrated persistent or recurrent hypercalcemia [99].

The use of Tc/Tl subtraction scintigraphy has been completely superseded by the superior results of 99mTc-sestamibi (MIBI) scanning with single-photon emission computed tomography (SPECT) imaging and is no longer in use today [100–102].

A series of 184 patients who underwent scan-directed UE after positive Tc/Tl subtraction or MIBI scanning were retrospectively followed for 59 months (range 6‑168 months). Following initial surgery, three individuals (1.6%) demonstrated persistent hypercalcemia. None of the patients developed recurrent hypercalcemia. Scan-directed UE represented a valid surgical strategy for a significant proportion of patients with PHPT, not leading to an increased incidence of persistent or recurrent hypercalcaemia [103].

Another modality that was studied to localize hyperfunctioning parathyroid glands is positron emission tomography (PET). The greater spatial and temporal resolution of PET compared to SPECT imaging should enable the detection of even the smallest pathological glands, which in theory could improve sensitivity [104].

One of the first reported uses of PET for parathyroid disease used 2‑deoxy-2-[18F]fluoro-D-glucose (18F-FDG), which revealed the location of a pathological parathyroid gland in a patient with PHPT [105].

In 1994, Hellman et al. [106] were the first authors to report the use of methionine (Met)-PET/CT to detect hyperfunctioning parathyroid tissue.

In a pilot study [107], 18F-fluorocholine PET/CT was recently shown to be a very promising new functioning imaging modality in localizing hyperfunctioning parathyroid glands in patients with PHPT.

A meta-analysis [108] that specifically compared the diagnostic accuracy of 11C-methionine and 18F-fluorocholine PET for parathyroid localization in patients with PHPT suggested the superiority of 18F-fluorocholine in terms of sensitivity, while the two tracers showed comparable accuracy in terms of positive predictive value. The pooled sensitivity of 18F-fluorocholine was higher than that of 11C-methionine (92% vs. 80%; *p* < 0.01), while the positive predictive value was similar (94% vs. 95%; *p* = 0.99).

The accuracy of 18F-fluorocholine PET/CT was higher than that of US and MIBI scintigraphy in comparing the three techniques. Its systematic use is discussed as the new first-line localization method in patients with PHPT [109].

## Practical advice before planning initial surgery

Percutaneous US can be used to examine the parathyroid and the thyroid gland in a single procedure. If the thyroid gland appears clinically normal but potentially treatable thyroid changes are diagnosed their surgical removal may be planned together with the enlarged parathyroid gland [110].

The use of US enables a size assessment of the enlarged parathyroid and differentiation between cystic and solid parathyroid lesions. In some cases, differentiation between thyroid and parathyroid adenoma, or the localization of multiple enlarged parathyroids, is imprecise or completely impossible. Providing information about the upper thoracic outlet is possible in experienced hands, but the technique does not yield information concerning deeper sections of the mediastinum.

Parathyroid scintigraphy with MIBI in the biphasic technique facilitates, primarily through SPECT imaging, not only the localization of one (or more) hyperfunctioning parathyroid gland enlargements, but also their location within the neck along with anatomical relations to other structures. The technique also makes it possible to localize ectopic hyperfunctioning glands in the mediastinum.

The CT and magnetic resonance imaging (MRI) play a minor role in parathyroid imaging but are important before reinterventions [111]. A meta-analysis of the English literature was performed to determine the collective sensitivity and specificity of MIBI in establishing its utility in directing limited explorations (LE). The average sensitivity and specificity of parathyroid scintigraphy were 90.7% and 98.8%, respectively, indicating its ability to guide an accurate limited (unilateral) procedure which could be realized in as many as 90% (MIBI sensitivity) of patients with solitary adenomas (87% of 6331 showed solitary adenoma) with a failure rate less than 1% [112].

Localization procedures are not strictly necessary prior to BNE; however, the true cost effectiveness of routine preoperative MIBI for patients with sporadic PHPT is primarily dependent upon the surgeon’s decision to use this information to proceed with less extended, more directed (limited) procedures, when appropriate [112].

Both US and MIBI (with SPECT) have become the first-line imaging procedures of choice before planning the surgical procedure.

The success of preoperative localization studies depends on the technique applied, the concomitant thyroid disease, the size and site of the enlarged parathyroid gland and on the investigator’s experience [113].

The surgeon should select the nuclear medicine facility for the localization examination. The precise interpretation and correlation of morphology (US) and function (scintigraphy) enables an accurate localization of hyperfunctioning parathyroid(s). After reviewing and discussing the localization results together with the nuclear medicine examiner, the endocrine surgeon plans the precise surgical approach.

Concordant positive localization using two imaging techniques is an optimal prerequisite for successful limited exploration, but it is not a guarantee for the correct localization or diagnosis of single-gland disease before the initial procedure. It has been possible to suspect or definitively diagnose multiple gland disease preoperatively in only a very few patients [113, 114].

To compensate for the uncertainty of the preoperative localization examination, Irvin [115] suggested the intraoperative use of a quick version of a modified PTH assay (with 10 min turnaround time) as an intraoperative adjunct to confirm that no other hyperfunctioning tissue is present and therefore predicting cure (“biochemical frozen section”) and to confirm the completeness of parathyroid resection [116]. The need for intraoperative PTH (IOPTH) monitoring as an intraoperative adjunct will depend upon the results of preoperative imaging and the nature of the surgical approach.

When preoperative localization with two imaging studies (US and MIBI or 18F-fluorocholine -PET-CT) is concordant for SGD, the use of IOPTH (monitoring) is under discussion. Some authors do not find IOPTH absolutely necessary; however, failure must be expected at between 5.0–7.5% comparing patients treated with and without IOPTH monitoring [117, 118]. If preoperative imaging is positive in only one imaging or discordant in two techniques, IOPTH monitoring is strongly recommended as soon as a targeted [focused] approach” (TE [FE]) is sought (Table 5). As shown in a recently published systematic review and meta-analysis, the use of IOPTH was associated with higher cure rates for patients with PHPT undergoing TE, TE without IOPTH was associated with less conversion to BNE at initial surgery but with lower cure rates and an increased risk of reoperation [119].

Until the beginning of the 1990s, BNE was the undisputed method of choice (gold standard), with a high postoperative success rate of 99.5% and a very low complication rate (0.3% permanent hypocalcemia; 0.8% persistent vocal cord paralysis; 0.3% mortality) in the hands of highly experienced endocrine surgeons [34]. The switch from a well-established surgical strategy to a method with which little experience had been gathered required critical evaluation.

The prerequisites for a change were the improved localization methods and the assurance of complete removal of the hyperactive parathyroid tissue despite LE through IOPTH measurements.

The shift of surgical strategy took place between 1990 and 2000 under the influence of endoscopic surgical techniques applied in different body regions (e.g., gallbladder, appendix, lungs etc.) and endocrine organs (thyroid, parathyroid, adrenals, pancreas) [120, 121] to minimize surgical access and surgical trauma, postoperative pain and complications. There was definitively a change from non-image-guided extended BNE to image-guided LE with SP [122].

Four prospective randomized trials [123–126] compared extended BNE with LE (UNE or TE [FE]) open exploration and SP and yielded identical postoperative results concerning function (persisting hypercalcemia, hypocalcemia) and patient satisfaction.

Patients undergoing a UE had a lower incidence of biochemical and severe symptomatic hypocalcemia in the early period following surgery compared with patients undergoing BNE [124]. The use of TE by open minimal incision technique using local anesthesia reduced operating times and caused less postoperative biochemical hypocalcemia compared with BNE [123].

Scan-directed UNE for PHPT did not significantly increase the incidence of persistent hypercalcemia compared with standard bilateral surgery [125].

The TE/FE and conventional parathyroidectomy were safe and effective. In addition, TE/FE had several advantages, including less postoperative pain, lower analgesic request rates, lower analgesic consumption, shorter scar lengths and better cosmetic satisfaction rates over a short time period [126].

The identical results of the four studies led to the conclusion that in sporadic PHPT with localized SGD, limited UE with identification and assessment of two ipsilateral (the prelocalized enlarged and one normal) glands or a TE exploration of the prelocalized gland and SP with IOPTH may become the new standard.

The terms TE and FE cover a large spectrum of different techniques (Table 4): surgery may apply a direct (cervical) access. By definition, the prelocalized parathyroid gland is exposed and SP is performed through a (short; 15–20 mm) skin incision in the middle of the neck. This may be done openly (by a mini-incision; OMIP [121, 127]), by means of video (endoscopic)-assisted (VAP) [128] or total endoscopic procedure (EP) [129–132]. OMIP may be performed under local anesthesia [133] and VAP in cervical block anesthesia [134].

A prospective randomized study compared conventional BNE and VAP. The latter was associated with shorter operating time, better cosmetic results and less painful postoperative courses. No cases of persistent PHPT presented in either group [128]. Both OMIP and VAP offered a valuable approach to solitary parathyroid adenoma with a similarly excellent success rate and a minimal morbidity rate, lower pain intensity within 24 h following surgery, lower analgesia request rate, lower analgesic consumption, shorter scar length, better physical functioning and bodily pain aspects of quality of life on early recovery and higher early cosmetic satisfaction rate in a prospective, randomized, blinded trial; however, these advantages of VAP were achieved at higher costs because of endoscopic tool involvement [135].

Surgeons experienced in conventional bilateral or unilateral open explorations have a short learning curve in OMIP, while the endoscopic techniques require various endoscopic equipment and more interventions to gain sufficient experience (longer learning curve).

The OMIP can also be performed with low morbidity rates in combination with unilateral thyroid lesions, if indicated [110]; however, the hyperactive, enlarged parathyroid gland cannot be reliably localized in all patients with sporadic PHPT [113]. Therefore, TE/FE is only possible and successful in selected patients.

In an early prospective single center study [127] it was documented that 83/100 (83%) patients with localized SGD (irrespective of additional ipsilateral thyroid disease) were selected for OMIP. Finally in 69 patients, OMIP was successfully finished, 9 of these had had previous neck surgery, and another 24 underwent additional ipsilateral thyroid resection. Permanent normocalcemia was achieved in 67 (97.1%) of 69 patients [127].

With high-quality imaging (MIBI and US) and IOPTH monitoring, OMIP (suitable for this technique were 627/1361 (36%) patients) could be performed with an equal cure rate (97%) as the standard cervical exploration, with no present evidence of delayed recurrence [136].

A consensus has developed that direct cervical access with TE/FE applying OMIP is the new gold standard and should be adopted for the majority of patients with sporadic PHPT and well-localized SGD [137]. The more direct the access, the less invasive the dissection. Therefore, surgical techniques allowing direct access to the enlarged parathyroid gland minimize the trauma of surgical exposure and are followed by less pain and by excellent cosmetic results. These techniques should be preferred. They may be alternatively performed with the patient under local anesthesia or cervical block anesthesia. Should preoperative imaging be incorrect (and the exploration to be extended to the contralateral side), extending incisions does not result in excessively long incisions (scars). Only techniques that meet these criteria should be termed minimally invasive [138].

As shown by Hargitai et al. [139], SP may be performed in selected cases with remote accesses. These techniques are performed with skin incisions followed by scars located outside the neck. They generally use two or more trocars applied through short (5–10 mm) incisions. Long working canals from the incision area(s) (axilla, region above the areola, mouth) are usually necessary to reach the parathyroid region, resulting in extensive soft tissue trauma. These techniques facilitate SP but not direct access to the prelocalized enlarged parathyroid gland. The assumption is false that simply shortening the surgical incision followed by an excellent cosmetic scar is sufficient to define a surgical procedure as minimally invasive [138].

Therefore, surgical procedures using an extracervical “remote access (with/without insufflation of CO_2_, anterior thoracic approach, transaxillary approach, axillary-breast approach, retroauricular, transoral sublingual and vestibular, vestibular transoral approach [139]) should not be referred to as minimally invasive. With or without robotic support, these endoscopic techniques call for advanced surgical endoscopic expertise and cause overall higher costs due to the necessary equipment than direct accesses with an open procedure, while sometimes requiring longer operating time.

## Special situations

### Ectopic parathyroid adenoma

In approximately 20%, the enlarged parathyroid glands may have an ectopic position, especially in the superior or inferior/anterior mediastinum and less commonly in the inferior/middle or inferior/posterior mediastinum.

When an ectopic mediastinal parathyroid adenoma is located in the superior or inferior/anterior mediastinum, it is often within the thyro-thymic ligament or thymus itself and may be removed by a transcervical approach achievable through a standard cervical approach (transcervical thymectomy; Table 4), as summarized earlier in history [31].

Parathyroid tumors localized either low inferior in the anterior mediastinum, in the inferior/middle or inferior/posterior mediastinum are relatively rare presentations documented in only 1–2%. In these localizations, surgeons have traditionally utilized open approaches, such as sternotomy (transsternal mediastinal exploration; Table 4) or thoracotomy (open transthoracic exploration; Table 4), to achieve adequate exposure to correctly localize, explore and safely remove the parathyroid gland. These approaches are associated with increased postoperative pain, a prolonged hospital stay and a higher rate of complications.

As described previously [49], Churchill and Cope in Boston performed the first well-documented transsternal exploration in Charles M and successfully removed a parathyroid adenoma to the left of the inferior vena cava in 1932. In 1941, Cope [140] published the first larger single-center experience with mediastinal parathyroid tumors and, having no adequate imaging techniques available at the time, emphasized that a two-stage procedure was indicated: The second-stage exploration of the mediastinum was to be done only after thorough neck exploration and reconfirmation of the diagnosis with careful biochemical testing. This recommendation was the undisputed rule until the mid-1990s.

In 1994, Prinz et al. [141] authored the first report of the endoscopic technique applied to remove an ectopic parathyroid adenoma using a transthoracic route. Video-assisted endoscopic techniques (with and without robotic assistance) have become indispensable for removing deeply ectopic, prelocalized, mediastinal parathyroid adenomas. Compared to the formerly applied transsternal approach, these techniques are less invasive, effective, comfortable and safe procedures in experienced hands and may be the procedure of choice in these selected situations [142, 143].

### Parathyroid carcinoma

In 1933, Sainton and Millot [144] described the first functional parathyroid carcinoma in the medical literature. They reported a malignant eosinophilic tumor of the parathyroid gland occurring in a patient with bone disease attributed to OFC associated with clinical and biochemical evidence of severe HPT.

Parathyroid carcinoma represents a rare malignancy of the parathyroid glands, which most often arises sporadically, accounts for less than 1% of PHPT cases and occurs equally in men and women. Loss of tumor suppressor function, frequently through *HRPT2/CDC73* mutations, drives disease progression by promoting excessive hormone secretion and aggressive local invasion [145].

Clinically, suspicion increases with the presence of a palpable lesion accompanied by severe hypercalcemia and markedly elevated PTH levels.

Histological diagnosis is sometimes difficult. The identification of capsular or vascular invasion may be initial signs and the loss of parafibromin assists in distinguishing malignant from benign lesions. Malignancy is often only confirmed by the emergence of locoregional or distant metastases during follow-up.

Extended open exploration with en bloc surgical resection of the tumor and regional lymph nodes remains the primary treatment and offers the best chance for long-term survival [146].

Prognosis depends largely on early complete resection and TNM stage at the time of surgery [146]. The 5‑year survival rates as pooled from different registries and case series are in the range of 76–85% and 10-year survival of 49–77% [145]. Persistent and early recurrent hypercalcemia drives long-term morbidity and mortality. Both frequently complicate outcomes and necessitate systemic therapy or targeted medical management [147].

For patients with widespread metastatic or unresectable disease, newer targeted approaches, such as tyrosine kinase inhibitors (TKIs), temozolomide and immune checkpoint inhibitors (ICIs), may offer clinical benefit to specific patient cohorts.

To improve the outcome of this rare malignancy, it is recommended that patients with parathyroid cancer should be managed in centers of excellence [148].

## Conclusion

The introduction of autoanalyzers into laboratory medicine has simplified and reduced the cost of laboratory tests. Serum calcium measurements are inexpensive and should be ordered routinely as part of any routine blood testing, regardless of the reason for the laboratory examination. In these tests, hypercalcemia is documented incidentally more often than expected. A careful differential diagnosis of hypercalcemia reveals PHPT as the cause in at least one third of cases. Disturbance of parathyroid metabolism (PHPT) is biochemically characterized by hypercalcemia, elevated or high-normal PTH, and high-normal or significantly increased calcium excretion in a 24‑h urine collection. In contrast to the past, PHPT currently presents with minimal or no symptoms. If sporadic PHPT is biochemically confirmed, definitive treatment, i.e. surgical normalization of the hyperactive parathyroid tissue (in the majority by selective extirpation of a solitary parathyroid adenoma), should be carefully considered in selected patients by a direct (minimally open) cervical access.

## Footnotes

1 Fuller Albright, as part of his medical training and research, spent time in Vienna from 1928 to 1929, where he worked with Jakob Erdheim. Albright deeply admired Erdheim’s expertise in human disease, later remarking that Erdheim “knew more about human disease than any other living man”.
